# 4,6-Dimethyl­pyrimidin-2(1*H*)-one–urea–water (1/1/1)

**DOI:** 10.1107/S1600536808020928

**Published:** 2008-07-16

**Authors:** Feng Wu, Yun-Qian Zhang, Qian-Jiang Zhu, Sai-Feng Xue, Zhu Tao

**Affiliations:** a Key Laboratory of Macrocyclic and Supramolecular Chemistry of Guizhou Province, Guizhou University, Guiyang 550025, People’s Republic of China; b Institute of Applied Chemistry, Guizhou University, Guiyang 550025, People’s Republic of China

## Abstract

In the crystal structure of the title compound, C_6_H_8_N_2_O·CH_4_N_2_O·H_2_O, mol­ecules are linked *via* N—H⋯O, O—H⋯N and O—H⋯O hydrogen bonds, forming a three–dimensional framework.

## Related literature

For general background, see: Zhao *et al.*, (2004 ▶); Zheng *et al.*, (2005 ▶).
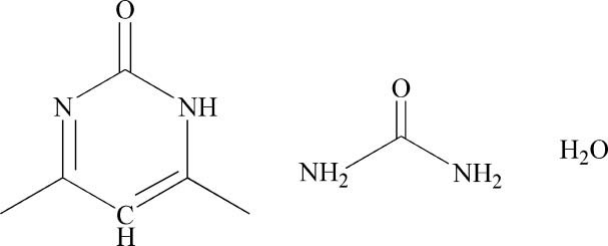


## Experimental

### 

#### Crystal data

C_6_H_8_N_2_O·CH_4_N_2_O·H_2_O
*M* *_r_* = 202.22
Triclinic, 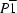

*a* = 8.1246 (5) Å
*b* = 8.4062 (5) Å
*c* = 8.9268 (9) Å
α = 105.007 (3)°
β = 103.857 (3)°
γ = 114.379 (2)°
*V* = 493.05 (7) Å^3^
*Z* = 2
Mo *K*α radiation
μ = 0.11 mm^−1^
*T* = 293 (2) K
0.22 × 0.16 × 0.11 mm

#### Data collection

Bruker APEXII CCD area-detector diffractometer
Absorption correction: multi-scan (*SADABS*; Bruker, 2005 ▶) *T*_min_ = 0.977, *T*_max_ = 0.998
5424 measured reflections
1728 independent reflections
1439 reflections with *I* > 2σ(*I*)
*R*_int_ = 0.019

#### Refinement

*R*[*F* ^2^ > 2σ(*F* ^2^)] = 0.053
*wR*(*F* ^2^) = 0.170
*S* = 1.10
1728 reflections
143 parameters
1 restraint
H atoms treated by a mixture of independent and constrained refinement
Δρ_max_ = 0.45 e Å^−3^
Δρ_min_ = −0.36 e Å^−3^

### 

Data collection: *APEX2* (Bruker, 2005 ▶); cell refinement: *SAINT* (Bruker, 2005 ▶); data reduction: *SAINT*; program(s) used to solve structure: *SHELXS97* (Sheldrick, 2008 ▶); program(s) used to refine structure: *SHELXL97* (Sheldrick, 2008 ▶); molecular graphics: *ORTEP-3* (Farrugia, 1997 ▶); software used to prepare material for publication: *WinGX* (Farrugia, 1999 ▶).

## Supplementary Material

Crystal structure: contains datablocks global, I. DOI: 10.1107/S1600536808020928/rk2094sup1.cif

Structure factors: contains datablocks I. DOI: 10.1107/S1600536808020928/rk2094Isup2.hkl

Additional supplementary materials:  crystallographic information; 3D view; checkCIF report

## Figures and Tables

**Table 1:** Hydrogen-bond geometry (Å, °)

*D*—H⋯*A*	*D*—H	H⋯*A*	*D*⋯*A*	*D*—H⋯*A*
N2—H2⋯O2	0.92 (3)	1.87 (3)	2.779 (2)	172 (3)
N3—H3*A*⋯O1	0.87 (3)	2.09 (3)	2.953 (3)	169 (3)
N3—H3*B*⋯O1^i^	0.83 (3)	2.16 (3)	2.896 (3)	149 (3)
N4—H4*A*⋯O2^ii^	0.93 (3)	2.04 (3)	2.968 (3)	174 (3)
N4—H4*B*⋯O1*W*^i^	0.83 (3)	2.12 (4)	2.948 (3)	175 (3)
O1*W*—H1*WA*⋯N1	0.850 (11)	2.121 (15)	2.930 (2)	159.1 (13)
O1*W*—H1*WB*⋯O1^iii^	0.850 (11)	2.209 (11)	3.009 (3)	156.9 (3)

Symmetry codes: (i) 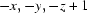; (ii) 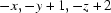; (iii) 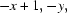 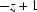.

**Fig. 1.**
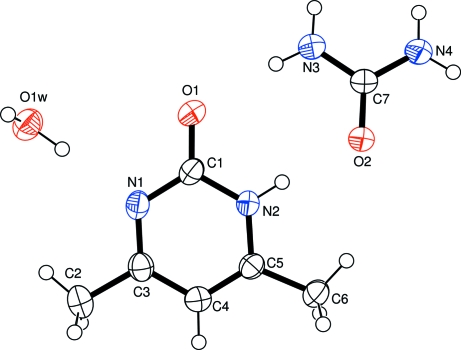
The molecular structure of I showing the atom–labelling scheme. Displacement ellipsoids are drawn at the 50% probability level. H atoms are presented as a small spheres of arbitrary radius.

**Fig. 2.**
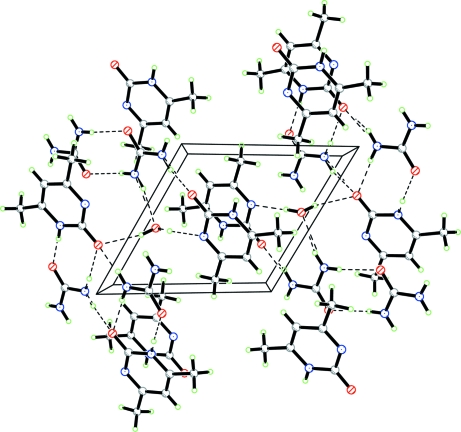
Packing diagram of I. H–bonds are shown as dashed lines.

## supplementary crystallographic information

### Comment

Recent years, we used different alkyl–substituted glycolurils as the building blocks to synthesize the partly alkyl substituted cucurbit[*n*]urils (Zhao *et al.*, 2004; Zheng *et al.*, 2005). In this work, we further report the crystal structure of a pyrimidine–substituted semi–glycoluril.

The crystal structure of the title compound C_6_H_8_N_2_O^.^CH_4_N_2_O^.^H_2_O, I, consists from 4,6–dimethylpyrimidin molecule, a urea molecule and a lattice water molecule. These molecules are linked *via* N—H···O, O—H···N and O—H···O hydrogen bonds forming a three–dimensional framework (Fig. 2).

### Experimental

In a 3–neck flask fitted with water knockout trap and the thermometer, (36 g, 0.6 mol) of urea dissolved in 100 ml of toluene, stirred vigorously, at room temperature. At the same time, (24 g, 0.24 mol) of acetylacetone was added into flask in one portion. The 1.5 ml of trifluoroacetic acid was added too in order to make the value of pH of the solvent is around 4. The reaction mixture was stired and heated and maintained at reflux for 4 h. After cooling to room temperature, the reaction mixture was filtered. The filtrate was concentrated by rotary evaporation at 318–323 K and then maintained overnight at room temperature and crystals of I appear (yield: 4.5 g, 0.036 mol, 15%)

### Refinement

Water H atoms and H atoms of amino group were located in a difference Fourier map and refined freely with *U*_iso_(H) = 1.2*U*_eq_(O, N). All H atoms based on C were placed in calculated positions and refined as riding, with C—H = 0.930–0.960Å with *U*_iso_(H) = 1.2–1.5 *U*_eq_(C).

### Crystal data

**Table:**

C_6_H_8_N_2_O·CH_4_N_2_O·H_2_O	*Z* = 2
*M**_r_* = 202.22	*F*_000_ = 216
Triclinic, *P*1	*D*_x_ = 1.362 Mg m^−^^3^
Hall symbol: -P 1	Mo *K*α radiation λ = 0.71073 Å
*a* = 8.1246 (5) Å	Cell parameters from 1728 reflections
*b* = 8.4062 (5) Å	θ = 2.6–25.1º
*c* = 8.9268 (9) Å	µ = 0.11 mm^−^^1^
α = 105.007 (3)º	*T* = 293 (2) K
β = 103.857 (3)º	Prism, colourless
γ = 114.379 (2)º	0.22 × 0.16 × 0.11 mm
*V* = 493.05 (7) Å^3^	

### Data collection

**Table:**

Bruker APEXII CCD area-detector diffractometer	1728 independent reflections
Radiation source: Fine–focus sealed tube	1439 reflections with *I* > 2σ(*I*)
Monochromator: Graphite	*R*_int_ = 0.019
*T* = 293(2) K	θ_max_ = 25.1º
φ and ω scans	θ_min_ = 2.6º
Absorption correction: multi-scan(SADABS; Bruker, 2005)	*h* = −9→9
*T*_min_ = 0.977, *T*_max_ = 0.998	*k* = −8→10
5424 measured reflections	*l* = −10→10

### Refinement

**Table:**

Refinement on *F*^2^	Secondary atom site location: Difmap
Least-squares matrix: Full	Hydrogen site location: Geom
*R*[*F*^2^ > 2σ(*F*^2^)] = 0.053	H atoms treated by a mixture of independent and constrained refinement
*wR*(*F*^2^) = 0.170	*w* = 1/[σ^2^(*F*_o_^2^) + (0.0859*P*)^2^ + 0.3229*P*] where *P* = (*F*_o_^2^ + 2*F*_c_^2^)/3
*S* = 1.10	(Δ/σ)_max_ < 0.001
1728 reflections	Δρ_max_ = 0.45 e Å^−^^3^
143 parameters	Δρ_min_ = −0.36 e Å^−^^3^
1 restraint	Extinction correction: None
Primary atom site location: Direct	

### Special details

**Table:**

Geometry. All s.u.'s (except the s.u. in the dihedral angle between two l.s. planes) are estimated using the full covariance matrix. The cell s.u.'s are taken into account individually in the estimation of s.u.'s in distances, angles and torsion angles; correlations between s.u.'s in cell parameters are only used when they are defined by crystal symmetry. An approximate (isotropic) treatment of cell s.u.'s is used for estimating s.u.'s involving l.s. planes.
Refinement. Refinement of F^2^ against ALL reflections. The weighted < i>R–factor wR and goodness of fit S are based on F^2^, conventional R–factors R are based on F, with F set to zero for negative F^2^. The threshold expression of F^2^ > σ(F^2^) is used only for calculating R–factors(gt) etc. and is not relevant to the choice of reflections for refinement. R–factors based on F^2^ are statistically about twice as large as those based on F, and R– factors based on ALL data will be even larger.

### Fractional atomic coordinates and isotropic or equivalent isotropic displacement parameters (Å^2^)

**Table:**

	*x*	*y*	*z*	*U*_iso_*/*U*_eq_	
C1	0.4613 (3)	0.3670 (3)	0.6702 (3)	0.0323 (5)	
C2	0.9295 (4)	0.6340 (4)	0.6375 (4)	0.0479 (7)	
H2A	0.9052	0.5182	0.5560	0.072*	
H2B	1.0477	0.6850	0.7341	0.072*	
H2C	0.9443	0.7254	0.5883	0.072*	
C3	0.7605 (3)	0.5927 (3)	0.6906 (3)	0.0343 (5)	
C4	0.7602 (3)	0.7390 (3)	0.8094 (3)	0.0342 (5)	
H4	0.8631	0.8644	0.8538	0.041*	
C5	0.6073 (3)	0.6940 (3)	0.8582 (3)	0.0304 (5)	
C6	0.5869 (4)	0.8321 (3)	0.9851 (3)	0.0393 (6)	
H6A	0.4664	0.7658	0.9983	0.059*	
H6B	0.5853	0.9287	0.9473	0.059*	
H6C	0.6958	0.8907	1.0913	0.059*	
C7	−0.0065 (3)	0.2768 (3)	0.8185 (3)	0.0326 (5)	
N1	0.6150 (3)	0.4123 (3)	0.6221 (2)	0.0345 (5)	
N2	0.4613 (3)	0.5103 (3)	0.7881 (2)	0.0305 (5)	
H2	0.361 (5)	0.477 (4)	0.825 (4)	0.037*	
N3	−0.0268 (4)	0.1313 (3)	0.6941 (3)	0.0456 (6)	
H3A	0.067 (5)	0.150 (4)	0.657 (4)	0.055*	
H3B	−0.135 (5)	0.035 (5)	0.631 (4)	0.055*	
N4	−0.1593 (3)	0.2454 (3)	0.8633 (3)	0.0411 (5)	
H4A	−0.151 (4)	0.352 (5)	0.938 (4)	0.049*	
H4B	−0.269 (5)	0.148 (5)	0.801 (4)	0.049*	
O1	0.3211 (2)	0.2007 (2)	0.6121 (2)	0.0431 (5)	
O2	0.1517 (2)	0.4327 (2)	0.8930 (2)	0.0383 (5)	
O1W	0.5570 (3)	0.0847 (2)	0.3512 (2)	0.0459 (5)	
H1WA	0.5959 (10)	0.175 (2)	0.445 (2)	0.055*	
H1WB	0.6047 (12)	0.0249 (14)	0.3918 (11)	0.055*	

### Atomic displacement parameters (Å^2^)

**Table:**

	*U*^11^	*U*^22^	*U*^33^	*U*^12^	*U*^13^	*U*^23^
C1	0.0355 (12)	0.0298 (12)	0.0289 (11)	0.0177 (10)	0.0113 (9)	0.0074 (9)
C2	0.0432 (14)	0.0535 (16)	0.0554 (16)	0.0269 (13)	0.0298 (13)	0.0205 (13)
C3	0.0341 (12)	0.0401 (13)	0.0342 (12)	0.0221 (11)	0.0143 (10)	0.0162 (10)
C4	0.0327 (12)	0.0300 (12)	0.0364 (12)	0.0140 (10)	0.0135 (10)	0.0117 (10)
C5	0.0341 (11)	0.0292 (11)	0.0289 (11)	0.0173 (10)	0.0116 (9)	0.0117 (9)
C6	0.0460 (14)	0.0297 (12)	0.0414 (13)	0.0186 (11)	0.0216 (11)	0.0103 (10)
C7	0.0318 (11)	0.0287 (12)	0.0343 (12)	0.0133 (10)	0.0123 (10)	0.0128 (9)
N1	0.0366 (10)	0.0378 (11)	0.0332 (10)	0.0227 (9)	0.0160 (8)	0.0113 (9)
N2	0.0304 (10)	0.0294 (10)	0.0310 (10)	0.0156 (9)	0.0135 (8)	0.0093 (8)
N3	0.0370 (12)	0.0340 (12)	0.0463 (13)	0.0084 (10)	0.0188 (10)	0.0023 (10)
N4	0.0302 (10)	0.0337 (11)	0.0488 (13)	0.0114 (9)	0.0167 (10)	0.0085 (10)
O1	0.0404 (10)	0.0296 (9)	0.0449 (10)	0.0129 (8)	0.0169 (8)	0.0025 (7)
O2	0.0328 (9)	0.0305 (9)	0.0433 (10)	0.0113 (7)	0.0184 (7)	0.0078 (7)
O1W	0.0477 (10)	0.0437 (10)	0.0396 (10)	0.0248 (9)	0.0124 (8)	0.0091 (8)

### Geometric parameters (Å, °)

**Table:**

C1—O1	1.244 (3)	C6—H6A	0.9600
C1—N1	1.356 (3)	C6—H6B	0.9600
C1—N2	1.379 (3)	C6—H6C	0.9600
C2—C3	1.495 (3)	C7—O2	1.249 (3)
C2—H2A	0.9600	C7—N4	1.339 (3)
C2—H2B	0.9600	C7—N3	1.341 (3)
C2—H2C	0.9600	N2—H2	0.92 (3)
C3—N1	1.329 (3)	N3—H3A	0.87 (3)
C3—C4	1.401 (3)	N3—H3B	0.83 (3)
C4—C5	1.354 (3)	N4—H4A	0.93 (3)
C4—H4	0.9300	N4—H4B	0.83 (3)
C5—N2	1.347 (3)	O1W—H1WA	0.850 (11)
C5—C6	1.492 (3)	O1W—H1WB	0.850 (11)
			
O1—C1—N1	122.19 (19)	C5—C6—H6B	109.5
O1—C1—N2	119.09 (19)	H6A—C6—H6B	109.5
N1—C1—N2	118.7 (2)	C5—C6—H6C	109.5
C3—C2—H2A	109.5	H6A—C6—H6C	109.5
C3—C2—H2B	109.5	H6B—C6—H6C	109.5
H2A—C2—H2B	109.5	O2—C7—N4	121.7 (2)
C3—C2—H2C	109.5	O2—C7—N3	120.9 (2)
H2A—C2—H2C	109.5	N4—C7—N3	117.3 (2)
H2B—C2—H2C	109.5	C3—N1—C1	118.88 (19)
N1—C3—C4	122.5 (2)	C5—N2—C1	123.10 (19)
N1—C3—C2	116.8 (2)	C5—N2—H2	118.8 (19)
C4—C3—C2	120.7 (2)	C1—N2—H2	117.9 (19)
C5—C4—C3	118.7 (2)	C7—N3—H3A	119 (2)
C5—C4—H4	120.6	C7—N3—H3B	122 (2)
C3—C4—H4	120.6	H3A—N3—H3B	116 (3)
N2—C5—C4	118.0 (2)	C7—N4—H4A	116.4 (19)
N2—C5—C6	116.75 (19)	C7—N4—H4B	119 (2)
C4—C5—C6	125.2 (2)	H4A—N4—H4B	120 (3)
C5—C6—H6A	109.5	H1WA—O1W—H1WB	96.3 (12)
			
N1—C3—C4—C5	1.1 (4)	O1—C1—N1—C3	−179.4 (2)
C2—C3—C4—C5	−177.7 (2)	N2—C1—N1—C3	0.3 (3)
C3—C4—C5—N2	−0.8 (3)	C4—C5—N2—C1	0.3 (3)
C3—C4—C5—C6	178.9 (2)	C6—C5—N2—C1	−179.4 (2)
C4—C3—N1—C1	−0.8 (3)	O1—C1—N2—C5	179.7 (2)
C2—C3—N1—C1	178.0 (2)	N1—C1—N2—C5	−0.1 (3)

### Hydrogen-bond geometry (Å, °)

**Table:**

*D*—H···*A*	*D*—H	H···*A*	*D*···*A*	*D*—H···*A*
N2—H2···O2	0.92 (3)	1.87 (3)	2.779 (2)	172 (3)
N3—H3A···O1	0.87 (3)	2.09 (3)	2.953 (3)	169 (3)
N3—H3B···O1^i^	0.83 (3)	2.16 (3)	2.896 (3)	149 (3)
N4—H4A···O2^ii^	0.93 (3)	2.04 (3)	2.968 (3)	174 (3)
N4—H4B···O1W^i^	0.83 (3)	2.12 (4)	2.948 (3)	175 (3)
O1W—H1WA···N1	0.850 (11)	2.121 (15)	2.930 (2)	159.1 (13)
O1W—H1WB···O1^iii^	0.850 (11)	2.209 (11)	3.009 (3)	156.9 (3)

Symmetry codes: (i) −*x*, −*y*, −*z*+1; (ii) −*x*, −*y*+1, −*z*+2; (iii) −*x*+1, −*y*, −*z*+1.
